# Research on the Composition and Distribution of Organic Sulfur in Coal

**DOI:** 10.3390/molecules21050630

**Published:** 2016-05-13

**Authors:** Lanjun Zhang, Zenghua Li, Yongliang Yang, Yinbo Zhou, Jinhu Li, Leilei Si, Biao Kong

**Affiliations:** 1 Key Laboratory of Coal Methane and Fire Control, Ministry of Education, China University of Mining and Technology, Xuzhou 221008, China; junjunzhang11@163.com (L.Z.); yyliang456@126.com (Y.Y.); zhouyinbo2011@163.com (Y.Z.); ljh911016@163.com (J.L.); si_leilei@163.com (L.S.); kongbiao8807@163.com (B.K.); 2 School of Safety Engineering, China University of Mining and Technology, Xuzhou 221116, China

**Keywords:** coal, XPS, organic solvent extraction, organic sulfur functional group

## Abstract

The structure and distribution of organic sulfur in coals of different rank and different sulfur content were studied by combining mild organic solvent extraction with XPS technology. The XPS results have shown that the distribution of organic sulfur in coal is related to the degree of metamorphism of coal. Namely, thiophenic sulfur content is reduced with decreasing metamorphic degree; sulfonic acid content rises with decreasing metamorphic degree; the contents of sulfate sulfur, sulfoxide and sulfone are rarely related with metamorphic degree. The solvent extraction and GC/MS test results have also shown that the composition and structure of free and soluble organic sulfur small molecules in coal is closely related to the metamorphic degree of coal. The free organic sulfur small molecules in coal of low metamorphic degree are mainly composed of aliphatic sulfides, while those in coal of medium and high metamorphic degree are mainly composed of thiophenes. Besides, the degree of aromatization of organic sulfur small molecules rises with increasing degree of coalification.

## 1. Introduction

S is one of the main harmful elements in coal, and SO_2_ emitted from the combustion of coal causes serious environmental pollution. Thus, research on the geochemical properties, distribution, form of occurrence, coal washing desulfurization and other aspects of sulfur have aroused people’s attention. Since the 1960s, some industrialized countries have enacted a lot of strict regulations and standards, limiting emissions of SO_2_ during the coal combustion process. These measures have greatly promoted the development of desulfurization technology. Sulfur in coal can be classified into two categories: inorganic sulfur and organic sulfur. Inorganic sulfur is present in the form of pyrite and sulfate, of which pyrite is the major inorganic sulfur form in most coals. Conventional coal washing technology can remove a large fraction of the inorganic sulfur in coal, but it has little influence on organic sulfur. Organic sulfur in coal, a collective term describing sulfur-containing organic functional groups, can be broadly divided into two types. One is sulfur-containing small molecule compounds which can be extracted and separated by using an organic solvent, while the other is attached to macromolecular skeleton forming C-S bonds and is difficult to separate from the coal matrix due to its complicated chemical structure and composition [1,2,3]. Organic sulfur in coal mainly includes the forms of mercaptans, disulphides, thioethers, sulfoxides, sulfones, thiophenes, sulfonates, *etc*. Because of the rather complicated structure of organic sulfur in coal, people are still unable to directly determine the content and form of organic sulfur in coal, and all kinds of analysis technologies cannot exactly describe the structure of organic sulfur in coal [4,5]. Therefore, research on its structure and composition remains a focus in the coal chemistry field all over the world. At present, XPS technology and solvent extraction are two major methods adopted in research on the structure of organic sulfur in coal. XPS technology, the most effective method of surface elemental analysis so far, is widely employed in the research on the sulfur forms of coal. It works by analyzing the elements (except for element H and He) existing on the sample surface and their modes of existence according to the different electron binding energy in the photoelectron spectra of different elements and valence states, and then it infers the composition and chemical structure of functional groups on the sample surface. This technology, due to its high identification power, good sensitivity, capability to maintain samples intact and other advantages in the speciation analysis of organic sulfur on coal surfaces, is the most effective coal surface analysis method available today [6,7,8]. Solvent extraction is one of the most important methods in studying coal structure. It releases soluble molecules in coal mainly by breaking intermolecular non-covalent bonds such as intermolecular H bonds and through the charge transfer, π-π bond, van der Waals force, electrostatic interactions, *etc*. From the conjoint analysis of solvent extraction and GC/MS, the existence as well as the separation and precipitation rules of each constituent in coal can be clearly recognized, resulting in advancement of coal structure research [9,10,11,12]. Mild solvent extraction methods can be used to study the composition and characteristics of sulfur-containing polycyclic and heterocyclic aromatic hydrocarbons of low molecular weight in coal, thus further obtaining relevant information about the organic sulfur small molecules. By selecting coal samples of different rank and different sulfur content and combining organic solvent mild extraction and XPS technology, this paper has comprehensively analyzed the structure of organic sulfur in coal to obtain important information such as the composition and distribution of organic sulfur functional groups in coal.

## 2. Results and Discussion

### 2.1. XPS Test Results and Analysis

**Figure 1:**
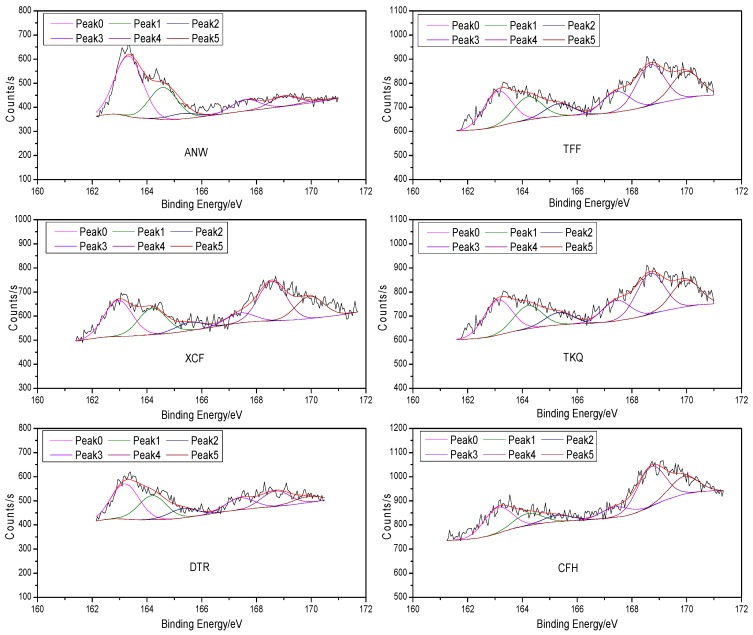
The XPS S 2p fitting curve of six raw coal samples.

**Figure 2:**
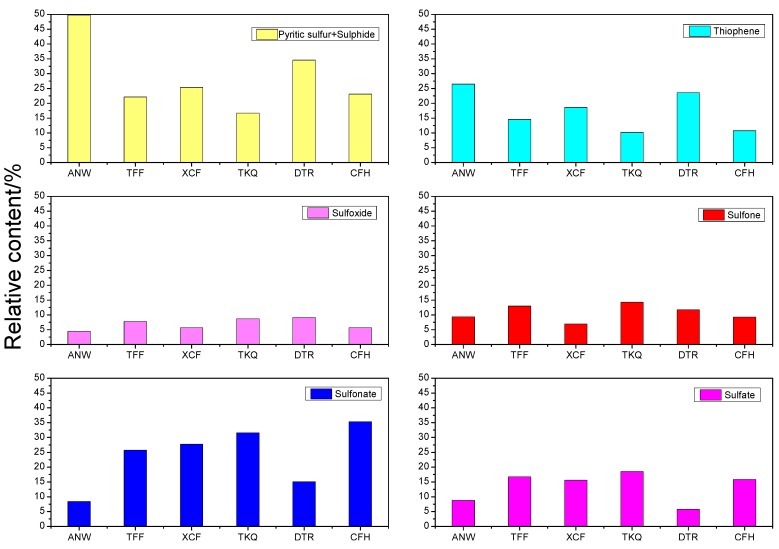
The relative content of different sulfur on six coal samples surface.

Figure 1 shows the XPS fitting curves of elemental S in six raw coals; Table 1 shows the XPS analysis results of the sulfur forms on the surface of six raw coal samples; Figure 2 is the contrastive analysis figures of the relative content of different sulfur forms in six raw coals. In the XPS spectra shown in Figure 1, the ordinate represents the strength of the electronic signal and the abscissa shows the electron binding energy. The elements existing on the sample surface as well as their existing forms can be analyzed through the electron binding energy, while their relative content can mainly be determined through the height and area of the fitting peaks. We can see from Figure 1 that six S 2p peaks can be fitted from the XPS spectrum of element S in each sample, wherein, the binding energy of Peak 0, 163.1 eV, is attributed to sulfide (mercaptan and thioether) sulfur and pyritic sulfur. At present, it is hard to distinguish mercaptan and thioether sulfide sulfur and pyritic sulfur using XPS technology because their binding energies are similar. Besides, the binding energy of Peak 1 (164.2 eV) belongs to thiophenic sulfur; Peak 2 (165.3 eV) to sulfoxide; Peak 3 (167.4 eV) to sulfone; Peak 4 (168.7 eV) to sulfonate; Peak 5 (169.9 eV) to sulfate. Due to the differences in the content, forms and distribution of organic sulfur and inorganic sulfur in various samples, their S 2p XPS spectra are also quite different.

It can be found in Table 1 and Figure 2 that after the removal of inorganic sulfur (iron sulfide and sulfate) from the six coal samples, among the organic sulfur left, sulfide sulfur, thiophenic sulfur and sulfonate have higher contents; sulfoxide and sulfone have lower contents. We can see from the results that the change law of sulfide sulfur and pyrite content with the degree of metamorphosis of the coal is not obvious (due to the interference of pyrite); thiophenic sulfur content is reduced with decreasing degree of metamorphism of coal; sulfoxide and sulfone content shows no obvious change rules; sulfonate content is raised with the decrease of metamorphic degree; and the content of sulfate sulfur is rarely related with metamorphic degree, with all XPS results of sulfate sulfur being significantly higher than the corresponding chemical analysis results. The relative content of iron sulfide sulfur and sulfide sulfur ANW sample reaches up to 49.83% in the XPS results and that of pyrite in this coal sample is 47.45% in the chemical analysis results, from which we can infer that the sulfide sulfur content of this coal is not high. The XPS analysis results show that the relative content of thiophenic sulfur in this coal is 26.49%, and the organic sulfur content of some other forms is relatively small.

Thus, it can be asserted that thiophenic sulfur is the main organic sulfur form of this coal. As both TFF coal and XCF coal come from Weng’an County, Guizhou Province, they are formed under similar geological conditions and have similar coal properties. Thus the distribution of various sulfur forms shown in their XPS analysis results is similar. The relative content of iron sulfide sulfur and sulfide sulfur in these two coals are 22.15% and 25.33%, respectively, in the XPS results, and those of pyrite in them are 33.04% and 44.77%, respectively, in the chemical analysis. Differences can be recognized by contrasting the XPS results with the chemical analysis results. The reason for this phenomenon is, on the one hand, pyrite on the surface of coal particles can be easily oxidized to sulfate, resulting in the corresponding decline in pyrite content. For example, the sulfate content of TFF coal rises from 0.58% in the chemical analysis results to 16.79% in the XPS results. On the other hand, XPS is a surface analysis technology which only detects the top 2–20 molecular layers on the coal surface, so XPS results are often quite different from phase analysis results. In addition, no dashing treatment has been conducted to the coal samples before the XPS tests, so the particle effect of minerals in coal will also have some interference on the test results [9,13,14]. It can also be seen from the results that the main organic sulfur form of TFF and XCF coal is thiophene; sulfide sulfur content is small; among the different oxidized forms of sulfur, the sulfonate content is highest, while the relative contents of sulfone and sulfoxide is lower. Among organic sulfur in TKQ coal, sulfonate accounts for the highest relative content, which is 31.61%. Since the chemical analysis results have shown that this coal only contains trace amounts of pyritic sulfur, the relative content of sulfide sulfur in this coal can be approximately regarded as 16.66%. The relative content of thiophenic sulfur, sulfone and sulfoxide shows a decreasing order. The XPS analysis results of sulfate sulfur are far higher than its chemical analysis results. This, in addition to the reasons mentioned above, may also result from large errors in the analysis results, because this coal has a low sulfur content of only 0.6%. The relative contents of iron sulfide sulfur and sulfide sulfur DTR coal is 34.58%, while the chemical analysis results show that pyrite is rarely contained in this coal. Therefore, it can be inferred that sulfide sulfur is the main organic sulfur form in this coal, followed by thiophenic sulfur; and the contents of sulfonic acid, sulfoxide and sulfone are the least. In the CFH coal sample, sulfonate accounts for the highest relative content, which is 35.35%, followed by sulfide sulfur and pyrite whose relative amount is 23.10% in total; the relative content of sulfate sulfur is as high as 15.85% which is far higher than the sulfate content obtained using the chemical analysis method; the relative content of thiophenic sulfur, sulfone, and sulfoxide is relatively scarce.

### 2.2. GC/MS Results and Analysis of THF Extract

GC/MS analysis of the extract was completed on an Agilent 6890/5975 Gas Chromatography and Mass Spectrometer (Agilent Technologies, Santa Clara, CA, USA) located at the Center of Modern Analysis of Nanjing University, with a DB-5 capillary column (30.0 m × 0.25 mm × 0.25 μm), the carrier gas being helium, flow rate of 1.0 mL/min, split ratio of 5:1, injection port temperature of 300 °C injection volume of 2 μL, ionization methods of E1, source ionization voltage of 70 eV, ion source temperature of 230 °C and mass (Ar) scan range of 30~650 amu. Computer retrieval and data comparisons are conducted according to the PBM method and the mass spectrometry data of compounds in the NIST chromatogram library, and then the structure of the compounds can be determined based on their confidence or similarity.

#### 2.2.1. Yangzhuang Lean Coal (YZS) from Huaibei

**Figure 3:**
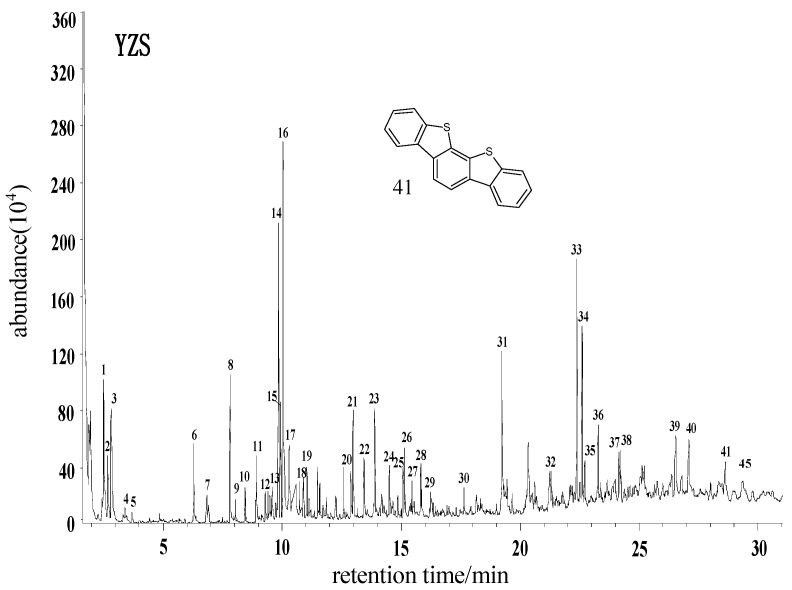
Total ion chromatogram (TIC) of THF extract of YZS coal samples.

Figure 3 is the total ion chromatogram (TIC) of THF extract of YZS coal samples. After the contrastive analysis of the chromatogram library, as shown in the Table 2, it can be determined that there are 41 kinds of compounds, as well as three compounds whose names cannot be determined, namely, Peaks 14, 27 and 28. From the composition of those compounds, it can be determined that aromatic hydrocarbons containing 1 to 6 rings represent the majority, compounds containing oxygen, nitrogen and sulfur also account for a certain proportion and the content of saturated aliphatic hydrocarbons is minimal, The mononuclear aromatics include 1 to 4 methyl-substituted compounds and phenol. Bicyclic aromatic hydrocarbons are composed of naphthalene and its methyl substituted derivatives, biphenyl and its methyl substituted compounds as well as diphenyl; tricycloarenes includes phenanthrene, its monomethyl and dimethyl substituted derivatives, phenylnaphthalene and fluorene; tetracycloarenes mainly include triphenylene and its methyl substituted derivatives, fluoranthene and perylene; pentacycloarenes comprise benzotetraphene and its methyl substituted derivatives; hexacycloarenes include naphthotetraphene and other substances. Oxygen-containing compounds mainly include esters, ketones and phenols; amide is the major nitrogen-containing compound; Peak 41, dibenzobenzodithiophene, is the only sulfur-containing compound detected.

#### 2.2.2. Taifeng Fat Coal (TFF) from Guizhou

**Figure 4:**
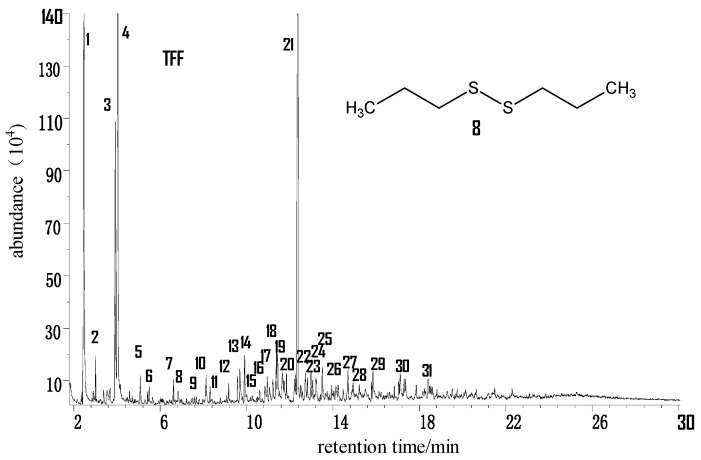
Total ion chromatogram (TIC) of the THF extract of TFF coal samples.

Figure 4 is the total ion chromatogram (TIC) of the THF extract of TFF coal samples. After the contrastive analysis of the chromatogram library, as shown in the Table 3, it can be determined that there are 31 kinds of compounds. Compared with YZS coal, the type and number of rings of aromatic hydrocarbons are less, with only 1- to 3-cycloarenes detected. In the extract, monocycloarenes included two kinds of trimethylbenzene derivatives and a kind of methyl- and butyl-substituted phenol; the bicycloarenes contained more kinds, including naphthalene and its alkyl-substituted compounds; tricycloarenes were mainly composed of phenanthrene, its monomethyl- and dimethyl-substituted derivatives as well as methyl fluorene. The alicyclic hydrocarbons are mainly *n*-alkanes covering the range from C_11_ to C_17_ (with the exception of C_15_). Besides, the cycloalkane trimethylcyclopentane is also detected. Oxygen-containing compounds include furan and phenol; the only sulfur-containing compound detected is dipropyl disulphide.

#### 2.2.3. Chifeng Lignite (CFH) from Inner Mongolia

**Figure 5:**
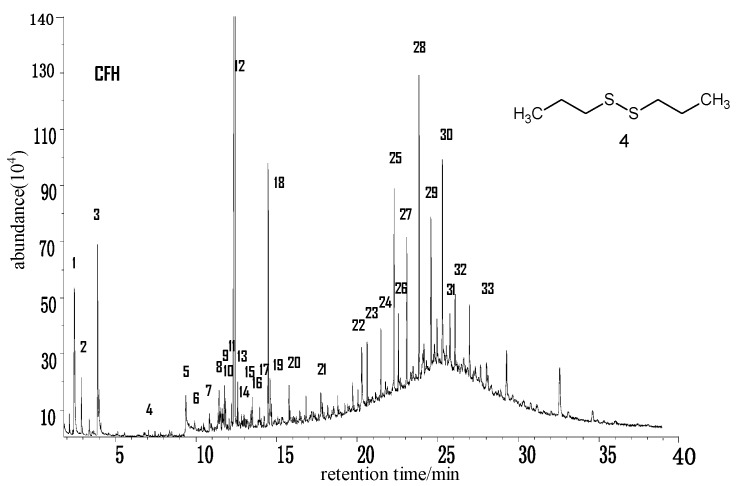
Total ion chromatogram (TIC) of the THF extract of CFH coal samples.

Figure 5 is the total ion chromatogram (TIC) of the THF extract of CFH coal samples. After the contrastive analysis of the chromatogram library, as shown in the Table 4, it can be determined that there are 30 kinds of compounds, as well as three compounds whose names cannot be determined, namely, Peaks 9, 11 and 20. Aromatic hydrocarbons, aliphatic hydrocarbons and heteroatom compounds are detected. Compared with the above two samples, less kinds of aromatic hydrocarbons are detected in CFH coal and most of them are bicycloarenes. In the monocycloarene class, only methyl- and butyl-substituted phenol and methylbenzoic acid are detected; similar the bicycloarene in the above two samples, those in CFH coal mainly include naphthalene and its mono- to tetramethyl-substituted compounds, biphenyl, indene, *etc*.; the only tricycloarene detected is *N*-phenylnaphthalene-2-amine; the only tetracycloarene detected is quaterphenyl. Aliphatic hydrocarbon levels are mainly high and medium length carbon chain *n*-alkanes which continuously distribute from C_23_ to C_31_. There are many kinds of oxygen-containing compounds, including furans, alcohols, ketones, acids, phenols, *etc*.; N phenylnaphthylamine is the only nitrogen-containing compound detected; the only sulfur-containing compound is dipropyldisulphide.

### 2.3. Discussion

The XPS results show that among the organic sulfur compounds in the coal samples, the content of sulfide (mercaptans and thioether) sulfur, thiophene and sulfonate sulfur is higher, while the content of sulfoxide and sulfone sulfur is lower; the distribution of organic sulfur in coal is related to the degree of metamorphosis of the coal. The change rules of sulfide (mercaptan and thioether) sulfur and pyrite content with the metamorphic degree of coal is not obvious (due to the interference effect of pyrite); thiophene sulfur content is reduced with decreasing degree of coal metamorphosis; sulfoxide and sulfone content shows no obvious change rules; sulfonate content is raised with the decrease of metamorphic degree; and the content of sulfate sulfur is rarely related with metamorphic degree. All the XPS results of sulfate sulfur were significantly higher than its corresponding chemical analysis results. Thiophenic sulfur is the major form of organic sulfur in ANW coal, and other organic sulfur content is relatively less. Thiophene is also main form of organic sulfur in the TFF and XCF samples; the content of sulfonate oxidation state is higher, while the contents of sulfide (mercaptan and thioether) sulfur, sulfone and sulfoxide are less. In TKQ coal, the sulfonate content is the highest, followed by sulfide (mercaptan and thioether) sulfur, while thiophenic sulfur, sulfone, and sulfoxide content are reduced in sequence. Organic sulfur in DTR coal shows a predominance of sulfide sulfur (mercaptan and thioether), while the content of thiophenic sulfur, sulfonate, sulfoxide and sulfone is less. In CFH, the relative content of sulfonate in the coal is the highest, followed by sulfide (mercaptan and thioether), while the thiophenic sulfur, sulfone, and sulfoxide contents are less.

Mild THF solvent extracts mainly contain free organic sulfur small molecules whose distribution and content are closely related to the degree of metamorphosis of the coal. That is, when the metamorphic degree of a coal sample is higher, there is a corresponding increase in the content ratio as well as kinds of aromatic hydrocarbons, while the relative content of aliphatic hydrocarbons drops; the chemical structure of extracts is simple with a small number of substituent groups; the content and kinds of heteroatom compounds are fewer. The carbon content of aliphatic hydrocarbons in extracts has a direct link to the metamorphic degree of coal, too. That is, with lower metamorphic degree of coal, the ratio of high-carbon alkanes in the extract grows higher. The free organic sulfur small molecules in coal of low metamorphic degree are mainly composed of aliphatic sulfides, while those in coal of medium and high metamorphic degree are mainly composed of thiophenes. Besides, the degree of aromatization of the organic sulfur small molecules rises with rising degree of coalification. Thiophenic sulfur was not detected in mild THF extracts of TFF and CFH coal samples, while the XPS results of TFF and CFH coal samples showed a certain proportion of thiophenic sulfur, which suggests that aliphatic hydrocarbon sulfides are more likely to be dissolved compared with thiophenic sulfur. It can also be seen from the aforementioned results that only a small number of free and soluble organic sulfur small molecules are detected in the extract. This is because most organic sulfur is attached to the macromolecular skeleton of coal by C-S bonds, making them hard to separate from the coal matrix, and leading to the small ratio of soluble organic matter. In addition, since GC/MS can only detect compounds with a relative molecular weight of less than 500, most of the soluble organic sulfur small molecules exceed the GC/MS detection range. Therefore, only very low amounts of organic sulfur small molecules can be detected.

## 3. Experimental Section

### 3.1. XPS Experimental Method and Coal Sample Selection

XPS measurement was conducted on an ESCALAB250 X-ray photoelectron spectrometer (Thermo Fisher, Waltham, MA, USA) at the Advanced Analysis and Computation Center of the China University of Mining and Technology. Al K alpha anode was employed; the power was 200 W, and the probe diameter was 900 μm. The full scanning transmission was 100 eV with step size being 1.000 eV; the narrow scanning transmission was 20 eV with step size being 0.05 eV; the basic vacuum was 10^−7^ Pa. C 1 s (284.6 eV) was taken as the calibration standard to conduct the check. In the XPS spectra, the ordinate represents the strength of the electronic signal, and the abscissa shows the electron binding energy. The S 2p XPS spectra obtained were peak fitted by using XPSPEAK 4.1 special software (Advanced Analysis & Computation Center of China University of Mining & Technology, Xuzhou, China), with the fitting parameters set as Shirley Background, 0% Lorentzian-Guaussian and FWHM 1.2 eV. Among the binding energies of sulfur compounds, 163.1 eV corresponds to sulfide sulfur (mercaptan and thioether) and pyritic sulfur, 164.2 eV to thiophenic sulfur, 165.3 eV to sulfoxide, 167.4 eV to sulfone, 168.7 eV to sulfonate, and 169.9 eV to sulfate [15,16,17,18].

Temperature, pressure, and time, the physical and chemical influence the degree of metamorphosis of the coal. In the coal metamorphism process, its physical characteristics, chemical composition and process performance undergo regular changes. According to the degree of coal metamorphosis, coal can be divided into three categories which are lignite, bituminous and anthracite. Six coal samples of different sulfur content and different metamorphic degree were selected as research objects. They were Anning anthracite (ANW) from Guizhou, Taifeng fat coal (TFF) from Guizhou, Xingcheng fat coal (XCF) from Guizhou, Tangkou gas coal (TKQ) from Shandong, Datong weakly caking coal (DTR) from Shanxi and Chifeng lignite (CFH) from Inner Mongolia, respectively. The bulk analysis, elemental analysis and analysis of various sulfur forms of the six coal samples are shown in Table 5 and Table 6. The proximate analyses and elemental analyses of the coal were in accordance with the Chinese national standards *GB/T* *30732-2014* and *GB/T 31391-2015*. The experimental samples were taken out from the sealed bags where they were stored, put into a 50 °C vacuum oven to dry for 12 h, and then cooled to room temperature. Two g of each dry coal sample was taken out and smashed to less than 74 μm to conduct the XPS tests.

Forms of sulfur in samples were analyzed in accordance with the Chinese national standard *GB/T215-2003, Determination of Forms of Sulfur in Coal*. The results are shown in Table 6. The rough contents of organic sulfur, iron sulfide sulfur and sulfate sulfur can be obtained with this method. According to Chinese national standard *GB/T15224.2-2004*, coals with different sulfur content are divided into six grades, namely special low sulfur coal with total sulfur less than or equal to 0.5%, low sulfur coal (0.51% to 1.00%), low and medium sulfur coal (1.01% to 1.50%), medium sulfur coal (1.51% to 2.00%), medium and high sulfur coal (2.01% to 3.00%) and high sulfur coal more than 3.00%. Based on the above criteria, TFF coal can be classified as high sulfur coal, XCF coal as medium and high sulfur coal, ANW coal and CFH coal as low and medium sulfur coals, and TKQ coal and DTR coal as low sulfur coals. Organic sulfur occupies a dominant position in low sulfur coal (TKQ coal and DTR coal), with very tiny content of other forms of sulfur. However, in addition to the predominant organic sulfur, there are also a certain amounts of iron sulfide and sulfate in medium-sulfur coal and high-sulfur coal (the other four samples).

### 3.2. THF Solvent Extraction Experimental Methods and Procedures

In the extraction experiments, tetrahydrofuran (THF) was selected as solvent, and Yangzhuang lean coal, Taifeng fat coal and Chifeng lignite were extracted by means of microwave assisted extraction. Taifeng fat coal and Chifeng lignite information has been introduced in above, that was not repeated here, Yang Zhuang thin coal coal quality analysis results are shown in Table 7. The extracts were analyzed by GC/MS to study the composition and structure of the organic sulfur small molecules in coal. The main instruments of the experiment include a CW-2008 multifunctional microwave reaction/extraction apparatus produced by Shanghai Xintuo Instruments Technology Co., Ltd. (Shanghai, China) and a RE-52AA rotary evaporator produced by Yarong Biochemical Instrument Factory (Shanghai, China). Forty g coal samples with particle size of 180~380 μm were taken out and put into the extractor, and then 400 mL tetrahydrofuran was added to the extractor. After the container was installed in accordance with the operating requirements, the circulating water was turned on. Then, the power supply of the equipment was started after making sure that experimental system was correctly connected. During the extraction, the pressure was set to normal pressure, the temperature to 50 °C and the extraction time to 4 h. After the end of extraction, suction filtration was applied to the coal and solvent mixtures in the container using a vacuum suction device, so as to separate the residual coal (coal samples after extraction) and extract. The RE-52AA rotary evaporator was used to concentrate the extract under the conditions of 30 r·min^−1^ and 60 °C until the extract was concentrated to 8 mL. Then, it was transferred to a sealed glass bottle for sealing and preservation and sent to the laboratory for GC/MS analysis.

## 4. Conclusions

In this paper, XPS analysis tests and a THF microwave assisted extraction method are adopted to study the structure of organic sulfur in coal. By conducting XPS analysis tests on coal of different sulfur content and different degrees of metamorphosis and in combination with GC/MS analysis of mild THF microwave extracts of the coal samples, this paper comprehensively analyzed the distribution rules of organic sulfur in coal. The following conclusions can be drawn:

(1) Among the organic sulfur in coal samples, the contents of sulfide (mercaptan and thioether) sulfur, thiophenic sulfur and sulfonate are higher, while the contents of sulfoxide and sulfone are lower. The change rule of sulfide sulfur (mercaptan and thioether) and pyrite content with the degree of metamorphosis of coal is not obvious (due to the interference of pyrite); thiophenic sulfur content is reduced with decreasing metamorphic degree of coal; sulfoxide and sulfone content shows no obvious change rule; sulfonate content is raised with the decrease of metamorphic degree; and the content of sulfate sulfur is rarely related with metamorphic degree.
(2) The distribution and content of free organic sulfur small molecules in coal are closely related to the degree of metamorphosis of coal. That is, with a higher metamorphic degree of a coal, the content ratio as well as the kinds of aromatic hydrocarbons in its extracts increase correspondingly, while the relative content of aliphatic hydrocarbons drops; the chemical structure of extracts is simple, with a small number of substituent groups; the content and kinds of heteroatomic compounds are lower. The content of aliphatic hydrocarbon compounds in extracts has a direct link to the metamorphic degree of coal, too. That is, with a lower degree of coal metamorphosis, the ratio of high-carbon number alkanes in the extract increases. The free organic sulfur small molecules in coal of low metamorphic degree are mainly composed of aliphatic sulfides, while those in coals of medium and high metamorphic degree are mainly thiophenes. Besides, the aromatization degree of organic sulfur small molecules rises with rising degree of coalification.

## Figures and Tables

**Table 1:** The XPS S 2p analysis of six raw coal samples.

Sample	Results	Pyritic Sulfur and Sulphide Sulfur	Thiophene	Sulfoxide	Sulfone	Sulfonate	Sulfate
ANW	Peak Area	320.05	170.15	28.88	60.36	53.99	8.80
	W(%)	49.83	26.49	4.50	9.40	8.41	1.37
TFF	Peak Area	186.07	122.45	65.42	109.03	216.06	141.01
	W(%)	22.15	14.58	7.79	12.98	25.72	16.79
XCF	Peak Area	191.86	141.07	43.48	52.70	210.35	118.01
	W(%)	25.33	18.62	5.74	6.96	27.77	15.58
TKQ	Peak Area	76.03	46.69	39.59	65.24	144.27	84.52
	W(%)	16.66	10.23	8.68	14.30	31.61	18.52
DTR	Peak Area	187.32	127.96	49.37	63.53	81.91	31.63
	W(%)	34.58	23.62	9.11	11.73	15.12	5.84
CFH	Peak Area	137.34	63.72	34.08	55.09	210.18	94.24
	W(%)	23.10	10.72	5.73	9.26	35.35	15.85

**Table 2:** YZS tetrahydrofuran extraction GC/MS analysis results.

Number	Name	Molecular Formula
1	1,4-Dimethylbenzene	C_8_H_10_
2	Ethenylbenzene	C_8_H_8_
3	γ-Butyrolactone	C_4_H_6_O_2_
4	1,2,3-Trimethylbenzene	C_9_H_12_
5	1,2,4-Trimethylbenzene	C_9_H_12_
6	Naphthalene	C_10_H_8_
7	1-(1,4-Dimethylcyclohex-3-en-1-yl)ethanone	C_10_H_16_O
8	1-Methylnaphthalene	C_11_H_10_
9	2-Methylnaphthalene	C_11_H_10_
10	2,6-Di-*tert*-butyl-4,4-dimethylcyclohexa-2,5-dien-1-one	C_16_H_26_O
11	Biphenyl	C_12_H_10_
12	1,6-Dimethylnaphthalene	C_12_H_12_
13	Diphenylmethane	C_13_H_12_
15	2,6-Di-*tert*-Butylcyclohexa-2,5-diene-1,4-dione	C_14_H_20_O_2_
16	(2a*R*,4a*R*,7a*S*,7b*R*)-2,2a,4a,7a-Tetramethyl-2a,3,4,4a,6,7,7a,7b-octahydro-5*H*-cyclopenta[cd]inden-5-one	C_15_H_22_O
17	4-Methylbiphenyl	C_13_H_12_
19	2,6-Di-*tert*-butyl-4-ethylphenol	C_16_H_26_O
19-1	9*H*-Fluorene	C_13_H_10_
19-2	4-*tert*-Butyl-2,6-di(propan-2-yl)phenyl acetate	C_18_H_28_O_2_
20	2,4,6-Tri-*tert*-butylphenol	C_18_H_30_O
21	3,5-Di-*tert*-butylbenzene-1,2-diol	C_14_H_22_O_2_
22	2,6-Di-*tert*-butyl-4-(hydroxymethyl)phenol	C_15_H_24_O2
23	Phenanthrene	C_14_H_10_
24	Butyl 2-methylpropyl benzene-1,2-dicarboxylate	C_16_H_22_O_4_
25	1-Methylphenanthrene	C_15_H_12_
26	2-Methylphenanthrene	C_15_H_12_
29	2-Phenylnaphthalene	C_16_H_12_
30	Hexadecanamide	C_16_H_33_C_18_H_35_NO
31	Octadec-9-enamide	C_18_H_35_NO
31-1	Triphenylene	C_18_H_12_
32	2-Methyltriphenylene	C_19_H_14_
33	4,4′-Ethane-1,2-diylbis(2,6-di-*tert*-butylphenol)	C_30_H_46_O_2_
34	Bis(2-ethylhexyl) decanedioate	C_26_H_50_O_4_
35	Benzo[pqr]tetraphene	C_20_H_12_
36	Benzo[j]fluoranthene	C_20_H_12_
37	10-Methylbenzo[pqr]tetraphene	C_21_H_14_
38	3-Methylperylene	C_21_H_14_
39	5,5′,6,6′,7,7′,8,8′-Octahydro-2,2′-binaphthalene	C_20_H_22_
40	Naphtho[7,8,1,2,3-nopqr]tetraphene	C_22_H_12_
41	Dibenzo[d,d′]benzo[1,2-b:5,4-b′]bisthiophene	C_18_H_10_S_2_
42	Naphtho[1,2,3,4-pqr]tetraphene	C_24_H_14_

**Table 3:** TFF tetrahydrofuran extraction GC/MS analysis results.

Number	Name	Molecular Formula
1	Tetrahydro-2-furanol	C_4_H_8_O_2_
2	1,1,3-Trimethylcyclopentane	C_8_H_16_
3	Dihydrofuran-2(3*H*)-one	C_4_H_6_O_2_
4	Tetrahydro-2-furanmethanol	C_5_H_10_O_2_
5	1,3,5-Trimethylbenzene	C_9_H_12_
6	1,2,4-Trimethylbenzene	C_9_H_12_
7	Undecane	C_11_H_24_
8	Dipropyl disulfide	C_6_H_14_S_2_
9	Naphthalene	C_10_H_8_
10	Dodecane	C_12_H_26_
11	Undecane,4,5-dimethyl-	C_13_H_28_
12	Tridecane	C_13_H_28_
13	2-Methylnaphthalene	C_11_H_10_
14	1-Methylnaphthalene	C_11_H_10_
15	2-Ethenylnaphthalene	C_12_H_10_
16	Tetradecane	C_14_H_30_
17	2-Ethylnaphthalene	C_12_H_12_
18	2,3-Dimethylnaphthalene	C_12_H_12_
19	1,7-Dimethylnaphthalene	C_12_H_12_
20	1,4-Dimethylnaphthalene	C_12_H_12_
21	2,6-di-*tert*-Butyl-4-methylphenol	C_15_H_24_O
22	2,3,6-Trimethylnaphthalene	C_13_H_14_
23	1,6,7-Trimethylnaphthalene	C_13_H_14_
24	1,4,6-Trimethylnaphthalene	C_13_H_14_
25	1,4,5-Trimethylnaphthalene	C_13_H_14_
26	Hexadecane	C_16_H_34_
27	Heptadecane	C_17_H_36_
28	2-Methyl-9*H*-fluorene	C_14_H_12_
29	Phenanthrene	C_14_H_10_
30	1-Methylphenanthrene	C_15_H_12_
31	2,5-Dimethylphenanthrene	C_16_H_14_

**Table 4:** CFH tetrahydrofuran extraction GC/MS analysis results.

Number	Name	Molecular Formula
1	Tetrahydro-2-furanol	C_4_H_8_O_2_
2	2-Methyl-2-pentenal	C_6_H_10_O
3	Dihydrofuran-2(3*H*)-one	C_4_H_6_O2
4	Dipropyl disulfide	C_6_H_14_S_2_
5	3-Methylbenzoic acid	C_8_H_8_O_2_
6	1-Methylnaphthalene	C_11_H_10_
7	Biphenyl	C_12_H_10_
8	2,3-Dimethylnaphthalene	C_12_H_12_
10	2,6-Di-*tert*-butylcyclohexa-2,5-diene-1,4-dione	C_14_H_20_O_2_
12	2,6-Di-*tert*-butyl-4-methylphenol	C_15_H_24_O
13	4,4,6,7-Tetramethyl-3,4-dihydronaphthalen-1(2*H*)-one	C_14_H_18_O
14	1,4,6-Trimethylnaphthalene	C_13_H_14_
15	1-Methyl-7-(propan-2-yl)naphthalene	C_14_H_16_
16	1,4,5-Trimethylnaphthalene	C_13_H_14_
17	5-*tert*-Butyl-1,1-dimethyl-2,3-dihydro-1*H*-indene	C_15_H_22_
18	1,6-Dimethyl-4-(propan-2-yl)naphthalene	C_15_H_18_
19	1,4-Dimethyl-6-(propan-2-yl)naphthalene	C_15_H_18_
21	3,8-Dimethyl-5-(propan-2-yl)naphthalen-2-ol	C_15_H_18_O
22	*N*-phenylnaphthalen-2-amine	C_16_H_13_N
23	Tricosane	C_23_H_48_
24	Tetracosane	C_24_H_50_
25	Pentacosane	C_25_H_52_
26	1-(2-{1-[(2-Ethylhexyl)oxy]ethenyl}phenyl)ethenol	C_18_H_26_O_2_
27	Hexacosane	C_26_H_54_
28	Heptacosane	C_27_H_56_
29	Octacosane	C_28_H_58_
30	Nonacosane	C_29_H_60_
31	1,1′:4′,1′′:4′′,1′′′-Quaterphenyl	C_24_H_18_
32	Triacontane	C_30_H_62_
33	Hentriacontane	C_31_H_64_

**Table 5:** The proximate and ultimate analysis of the six coal samples.

Sample	Proximate Analysis (wt % as Received)				Ultimate Analysis(wt % Daf)			
	M_ad_	A_ad_	V_ad_	FC_ad_	O_daf_	C_daf_	H_daf_	N_daf_
ANW	1.84	7.91	6.90	83.35	2.90	91.05	3.23	1.34
TFF	1.22	17.38	29.69	51.71	5.55	83.59	5.29	1.42
XCF	1.39	16.61	32.94	49.06	7.11	83.11	5.47	1.43
TKQ	2.32	9.83	34.85	53.00	10.21	82.24	5.18	1.63
DTR	2.86	7.90	21.80	67.44	8.94	85.59	4.08	0.74
CFH	15.32	13.89	31.88	38.91	18.58	74.63	4.47	0.86

**Table 6:** The sulfur forms of six coal samples.

Sample	S_t.d_/%	S_p.d_/%		S_s.d_/%		S_o.d_/%	
	Absolute	Absolute	Relative	Absolute	Relative	Absolute	Relative
ANW	1.37	0.65	47.45	0.01	0.73	0.70	51.09
TFF	3.42	1.13	33.04	0.02	0.58	2.27	66.37
XCF	2.39	1.07	44.77	0.03	1.26	1.29	53.97
TKQ	0.67	0.03	4.48	0.00	0.00	0.64	95.52
DTR	0.60	0.04	6.67	0.04	6.67	0.51	85.00
CFH	1.25	0.40	32.00	0.05	4.00	0.80	64.00

S_t.d_—total sulfur; S_p.d_—iron sulfide; S_s.d_—sulfate; S_o.d_—organic sulfur.

**Table 7:** The proximate and ultimate analysis of YZS coal samples.

Sample	Proximate Analysis (wt % as Received)				Ultimate Analysis (wt % Daf)			
	M_ad_	A_ad_	V_ad_	FC_ad_	O_daf_	C_daf_	H_daf_	N_daf_
YZS	1.58	46.16	5.30	40.86	2.50	89.0	4.12	3.3
